# Anæsthetics and Insanity

**Published:** 1899-11-18

**Authors:** 


### ANÆSTHETICS AND INSANITY.

A very interesting point was discussed by Dr. Savage at the last meeting of the Society of Anæsthetists, namely, the relation between the use of anæsthetics and the occurrence of insanity. He considered the subject from two points of view; first, taking anæsthetics as causes of insanity; and, next, considering their effects upon those already insane, or highly neurotic, or who had already suffered from attacks of insanity. Any drug, he said, which could produce temporary mental disorder might produce disorder of a more lasting nature. Anæsthetics producing delirium might set up mania, and such as produced insensibility might cause stupor and mental confusion. Nevertheless, anæsthetics rarely produced insanity except in patients who had had previous attacks, or were predisposed by exhaustion or allied states. Again, it must be noted that when an anæsthetic was given an operation was usually performed, and when insanity appeared to follow the administration of an anæsthetic it might really be due rather to the operation than to the drug. He did not think that the administration of one anæsthetic more than another was likely to cause mental disorder. He had seen mania follow the administration of gas for slight dental operations. When patients were the subjects of recurring attacks of insanity the administration of anæsthetics was associated with considerable danger, and the redevelopment of mental symptoms which were passing off might follow the administration of an anæsthetic to a convalescent. On the other hand, little or no danger resulted from the administration of anæsthetics to those who were already insane. Turning to the other side of the question, namely, the treatment of insanity by the administration of anæsthetics, he could only say that it had failed in all cases. Maniacal patients, though quieted for the time, returned to their maniacal excitement, the sleep produced by anæsthetics being temporary and of little service. Such administration, however, was harmless. The most interesting point insisted on by Dr. Savage seems to be the danger of administering anæsthetics to those who have previously suffered from attacks, or who are in a very unstable mental condition.

